# Supplementary material for: Oral characteristics and dietary habits of preterm children: A retrospective study using National Health Screening Program for Infants and Children
Source: PLoS One. 2023 Mar 2;18(3):e0281896. doi: 10.1371/journal.pone.0281896 (PMC9980808; doi:10.1371/journal.pone.0281896)
Supplement: Attachment — Submitted filename: response to reviewer_major revision.docx [file pone.0281896.s001.docx]

**A rebuttal letter to editor and reviewers**

Dear Editor and Reviewers,

We thank you for giving us the opportunity to submit a revised draft of manuscript and your thoughtful suggestions and insights. The manuscript has benefited from these insightful suggestions. I look forward to working with you and the reviewers to move this manuscript closer to publication in PLOS ONE. The manuscript has been rechecked and the necessary changes have been made in accordance with the reviewers’ suggestions.

Thank you for your consideration. I look forward to hearing from you.

Here is a point-by-point response to the reviewers’ comments and concerns. The reviewers’ comments are in bold. Our response follows and edited manuscript is highlighted in yellow.

**Additional Editor Comments:**

**1. Please ensure that your manuscript meets PLOS ONE's style requirements, including those for file naming.**

Thank you for your comment. We looked over the entire manuscript to ensure it meets PLOS ONE’s style requirements including file naming.

**2. Please remove any funding-related text from the manuscript and let us know how you would like to update your Funding Statement. We note that you have provided funding information that is not currently declared in your Funding Statement. However, funding information should not appear in the Acknowledgments section or other areas of your manuscript.** **Please remove any funding-related text from the manuscript and let us know how you would like to update your Funding Statement.** **Please include your amended statements within your cover letter; we will change the online submission form on your behalf.**

Thank you for your comment. We reorganized Acknowledgements, Funding and Data Availability section for more clarification-which is also related to your next comment (comment #3).

Acknowledgement

This part was originally written to pay special gratitude to Ji Young Park who helped the authors with raw data extraction from NHIS. NHIS information is private and not easily assessed and has many complicated steps of verifications for extracting data source, even for professionals. However, this is not related to funding. We have edited Acknowledgement section to be more specific and removed source of confusion.

The authors are especially grateful to Ji Young Park for assisting raw data collection from NHIS. Data collection from NHIS has many complicated steps of verifications and not easily assessed even for professionals.

Funding

All funding related information regarding this manuscript is written below.

This work was supported by the National Research Foundation of Korea(NRF) grant funded by the Korea government(MSIT) (No. NRF-2020R1G1A1100275) awarded to Chung-Min Kang. The funders had no role in study design, data collection and analysis, decision to publish, or preparation of the manuscript.

Data availability statement

The minimal data set underlying the results described in our manuscript was collected from data provided by National Health Screening Program for Infants and Children (NHSIC) of the National Health Insurance Service (NHIS) of Korea. This data cannot be shared with the public and only the verified researchers who meet the accessibility criteria has access in only permitted time. The authors had no privileges related to these criteria compared to others. The authors declare no conflict of interest with NHIS.

**3. In your Data Availability statement, you have not specified where the minimal data set underlying the results described in your manuscript can be found.**

Thank you for your thoughtful comment. We have edited and specified where the minimal data set underlying the results can be found in Data availability statement. Please refer to the previous comment for revised text.

**4.** **From Reviewer 1**: **It is quite evident that you need to defend your research methodology and statistical techniques; otherwise, you should revise them, accordingly**.
**From Reviewer 2:** **By the same token, Reviewer 2 had grave concerns with respect to your statistical analyses as Reviewer 1 held.**
**From Reviewer 3:** **As Reviewer 3 sharply pointed out, you need to be more careful about the validity and accuracy of statistics in your work.**
Thank you for your comment. We revised table 4 and added table 5 to improve comparing the dental experience of preterm birth and full-term birth. The average dental experience per 1 dental visit, the sum of each dental treatment per single dental visit, are added as the outcome variable. The dental experience and the average dental experience per 1 dental visit of those who had undergone an oral health screening at least once and those who had never had oral health screening are analyzed separately. By making these changes, we hope we have provided more clear research methodology and valid statistics. Reviewer 1,2 and 3 have commonly given comments about our methodology and statistics. We have edited our manuscript and written our responses to each reviewer in hopes of elucidating more precisely study design and results of our study.

**Reply to reviewer #1**

**1. In the research method, it is necessary to explain more accurately what items were investigated in this study. Currently, there are only survey contents, and it is necessary to describe what content was extracted as a key element and compared based on the answers of the survey.**

Our response: Thank you for your insightful comment. In order to explain more accurately what and why items in this manuscripts were selected, we first thought it would be better to explain in details where the minimal data set came from. Like explained in the minimal data set section, data for this manuscript was collected from data provided by National Health Insurance Service(NHIS). NHIS has nationwide infant medical and oral screening cohort data base user manual but is not provided in English. We have selected parts of the manual and included the original Korean version as well as the translated English (in blue font) for your better understanding of the format of data collection.

Nationwide Infant Medical and Oral Screening Cohort Database(DB) User Manual (ver 1.0)

NHIS Big Data Office

1. Introduction to Nationwide Infant Medical and Oral Screening Cohort DB

A. Definition of Nationwide Infant Medical and Oral Screening Cohort DB

Cohort DB provides **5% of screening data collected from total infants who completed first or second session of screening and are born between 2008~2012**

B. Nationwide Infant Medical and Oral Screening Cohort DB population design

1) Population : total infants who completed first or second session of screening

2) Population design : Infants born between 2008~2012 and 5% of each year’s population

3) Total population : **84005 (n)**

4) Collection method : Cohort database

5) Collection period : 2008~2015 (8 years)

The bolded part is written in “Study data” in “Methods” of the manuscript.

The available data provided by NHIS is 5% of infants born between 2008 and 2012 who underwent at least one of the first and second medical screening examinations (n=84,005).

Screening DB items

1. Health screening DB : 239 items

| Number | Variable | Variable Code | Variable type | Note |
| --- | --- | --- | --- | --- |
| 3 | Personal ID number | PERSON_ID | NUM(8) |  |
| 11 | [Q2] Was your child born preterm? | **I1Q**_PREM | NUM(8) | 1 Yes  2 No |
| 12 | [Q3] Birthweight of your child? | I1Q_BTH_WGHT | NUM(8) | kg |

| 210 | [Q2] Was your child born preterm? | **I7Q**_PREM | NUM(8) | 1 Yes  2 No |
| --- | --- | --- | --- | --- |
| 211 | [Q3] Birthweight of your child? | I7Q_BTH_WGHT | NUM(8) | kg |

These are examples of how the data is provided from NHIS database. There are in total 239 questions in health screening data. All the infants who complete screening are coded by personal ID number and personal information is unknown. Number 11 asks the parents answering the health screening survey if their child was born preterm in which their answer is coded as yes or no. Number 210 asks the same question but the different variable code is noticeable. I1Q mean 1st session and I7Q means 7th session; indicating there are same question asked every session.

Oral Screening DB items

1. Health screening DB : 98 items

| Number | Variable | English | Variable type | Note |
| --- | --- | --- | --- | --- |
| 3 | Personal ID number | PERSON_ID | NUM(8) |  |

0 : Erupted

1 : Unerupted

2 : Erupting (partial-eruption)

3 : Hypomineralization, decalcification

4 : Dental caries

5 : Treated

6 : Pit and fissure sealant

Oral health screening has 98 items. 0-6 lists how the present state of the child’s oral condition diagnosed by the dentist who completes the oral health screening is scored.

‘/userdata01/room005/data_source’;

| /userdata01 | room001 ~ room010 |
| --- | --- |
| /userdata02 | room011 ~ room020 |
| /userdata03 | room021 ~ room030 |
| /userdata04 | room031 ~ room040 |
| /userdata05 | room041 ~ room050 |
| /userdata06 | room051 ~ room060 |
| /userdata07 | room061 ~ room070 |
| /userdata08 | room071 ~ room080 |
| /userdata09 | room081 ~ room090 |
| /userdata10 | room091 ~ room100 |

The images above show how one assesses the NHIS database. The ID and password are only granted to permitted persons and there are many complicated steps for verification, even for professionals. NHIS database is accessed in virtual SAS software by VMware Horizon port.

The extracted data are organized by category and room number and there is one ID and password for each room and can only be assessed by one VPN server IP. Permitted data source can only be outputted to a folder in virtual software desktop and only be open in virtual software. Every data room has finite time for opening access and if one wishes to reopen the data, one must go through whole verification process again to gain virtual ID and password.

Database is set-up based on screening questions and provided only by the code (or numeric answer for example, birthweight). Our retrospective study was based on this cohort DB. We have summarized and added this part in “Study data” in “Methods” for readers to be more clear of where our data came from.

Revised text: Data is analyzed by remote access to a virtual computer only for a certain period of permitted time. The infants who complete screening are coded by personal identification number and saved in the database. There are 239 items in health screening and 98 items in oral screening database.

**2. In addition, if it’s a true longitudinal study, you should follow-up the patient during the observation period to observe oral health and changes in lifestyle that can affect it. Alternatively, different samples should be investigated on the premise that the population is the same at different points in time so that they can know the change from year to year. In the current study, it is difficult to say that it is a true longitudinal study though it has period to increase the number of samples.**

Our response: Thank you for your thoughtful comment. This database is longitudinally constructed based on whether a child received the first or second medical screening. Therefore, as the follow-up time increases, there are more missing values because national medical and oral health screenings are provided but not mandatory. Since there were numerous missing values, our analytic methods mainly performed the Chi-square test at each given time point that screening was provided. If this database contains longitudinally followed-up data, with all children receiving the whole series of seven medical and three oral health screening services, we could have chosen other methods, such as survival analysis.

It is impossible to follow-up the same patient group during the observation period by NHIS policy. NHIS does not allow individual verification, the only available data accessible to authors is organized by the oral examination age group. Regarding the true longitudinal study, our study involves follow-up of children who have either completed first or second medical check-up. There are fewer patients who complete medical and/or dental check-up as they age, and thus it is difficult to follow-up the same individual patient. We have added this part to our limits in our discussion.

Revised text: This data does not provide personal information but are only classified as person identification number and therefore impossible to follow-up the patient during the screening period. The dates of medical and oral screening sessions do not match and therefore is not easy to study data collected at different points in time. The inability to compare the results of the same period is another limitation.

**3. The only difference between the experimental group and the control group is the difference in perception of the parents' oral health at 42-53 months. There is a slight statistically significant difference. Why? should be dealt with in more detail in the consideration.**

Our response: Thank you for your comment. Tooth cavitation is usually the first visible sign for parents to recognize dental caries of their children. It takes months of time for a tooth to erupt through gingiva and for the crown to reach the functional occlusal surface. Thus visible caries can only be noticed by patients and/or parents only after some time after the beginning of tooth eruption. Primary teeth complete eruption in 24-30 months in average. The first oral health screening is done at 18-29 months and second at 42-53 months which is the most possible reason for preterm birth parents to report child’s poor oral hygiene at 42-53 months.

Revised text: It is difficult to observe caries at 18-29 months when primary molars start to erupt but have not fully erupted to the occlusal surface. Child’s oral hygiene becomes more noticeable to parents of preterm infants when tooth cavitation due to dental caries is recognized after the complete eruption of primary dentition. This is the underlying reason why preterm parents of 42-53 months significantly recognized the child’s oral hygiene to be poor. However, there was no significant difference between the two groups at the follow-up examination at 54-65 months.

**4. If the babies come to the dentist more often, more treatment will be done, and if there is no significant difference in oral condition, you may even conclude that full-term children's oral health is worse than preterm children. Since the questionnaire items of national screening are too simple, it is difficult to compare the information of the two groups. All explanations are not based on the results obtained in the current study, but are all guessed using previous studies. Changes in oral conditions and oral health habits according to age changes have not been studied.**

Our response: Thank you for your thoughtful comment. Like you mentioned in your comment, if there are more dental visits, more treatment can be done and this may come to a conclusion that full-term children’s oral is worse than preterm children. Thus, we have added analysis of number of dental treatments done per dental visit. There was a significant difference in number of dental visits between full-term and preterm children, however, number of dental treatments done per dental visit were not significantly different. This indicates that there is no significant difference in oral condition between two groups, especially if pre-term children made the effort to visit dental office for dental check-up and treatment. Table 4, 5, Figure 2 and the manuscript are edited accordingly.

Revised text: The percentage of visits to the dentist at least once were significantly higher in full-term group (75.8%) than preterm group (74.0%) (*p*=0.036) (Table 4). However, the average number of dental visits for children were not significant between full-term (5.94±5.43) and preterm groups (5.90±5.31) (*p* = 0.783). Full-term children underwent 1 visit pulpectomy, pulpotomy and rubber dam application more often than the preterm children and required behavior management for less than 15 minutes. Full-term children had more glass ionomer restorations than preterm children (*p*=0.007) and preterm children had more sealants performed (*p*=0.034) when comparing dental treatment performed per dental visit. Table 5 lists comparison of number of dental treatments done according to oral health screening completion. There was no difference in dental treatments between full-term and preterm children if a child had never received oral health screening. For preterm children who have completed at least one oral health screening, 1-visit pulpectomy (*p* = 0.007) and 2-visit pulpectomy (*p* = 0.042) were performed less. Dietary habits and dental experiences of preterm children are summarized in Fig 2.

**5. Now, I will ask the author who submitted the manuscript. First, why didn't you use the latest data?**
The latest data available for from NHIS database cohort is from 2008-2015, the study data included in this study. I have included a table depicting time line of medical and oral screening sessions. Medical screening commenced in 2008 and oral screening in 2009. The following data is also from NHIS database user manual explained previously in your comment #1.

|  | Session | Screening Years |
| --- | --- | --- |
| Medical  health screening | 1  2  3  4  5  6  7 | 2008-2013  2008~2014  2009~2015  2010~2015  2011~2015  2012~2015  2013~2015 |
| Oral  health screening | 1  2  3 | 2009~2015  2011~2015  2012~2015 |

**6. Second,** **why did you not attempt to interpret it in connection with oral condition data such as dmfs?**
Our response: Thank you for your thoughtful comment. We would also be delighted to interpret data more connected to oral condition such as dmfs however, this data is impossible to extract from the provided database. Mentioned in your comment #1, database only provides tooth diagnosis according to 0-6. Score 5 indicates treated tooth however, treated can include not only filling but also other types of dental treatments such as crown restorations. It is also difficult to know if a tooth is missing.

**7. Third, why didn't you study the data (year) collected at different points in time?**

Our response: Thank you for your comment. Unfortunately, Korean medical and oral health screening sessions do not match and therefore it is not easy to study data by year. Seven medical screening sessions are provided between the ages of 4 and 71 months: at 4–6, 9–12, 18–24, 30–36, 42–48, 54–60, and 66–71 months. Three oral screening sessions are provided: at 18–29, 42–53, and 54–65 months. We have added this part as a limitation of our study. It was stated that the inability to compare the results of the same period could be a limitation of the study. This is also related to your comment #2.

Revised text: The dates of medical and oral screening sessions do not match and therefore is not easy to study data collected at different points in time. The inability to compare the results of the same period is another limitation.

**8. Concern of the reviewer: The survey was only about the ratio and type of premature birth, analysis, and questionnaire, but I don’t think there’s any strong correlation, and there is lack of explanatory power to assess the results from citing other studies. Although it has the advantage of being the first nationwide research, it is not suitable for publication due to insufficient research contents. Please reinforce the contents after a major revision and submit it again.**
Our response: Thank you for your insightful comment. Please refer to your previous comments and our replies for detailed explanation of what we have done to make this manuscript more meaningful. We have added detailed comprehensive elucidation on where and how we extracted our raw data and the complicated process and the limits within. From this limited source, we have tried to pull out relevant variables and perform worthwhile analysis. We performed further analysis focusing on dentistry: we have looked at dental experience and treatment characteristics of preterm and full-term children and compared them per dental visit between two groups and also compared them based on completion of oral health screening.

We modified Table 4 and added Table 5 and re-edited figure 2 to make this manuscript more meaningful. The following statements are take home messages from our further analysis:

1. There were more pre-term children who have never visited dental office.

2. However, the average number of dental visits were similar between full-term (5.94) and preterm (5.90) groups.

3. If a preterm-born child received at least one oral health screening service, the number of pulpectomies aiming to treat advanced dental caries decreased.

4. Therefore should pre-term children make the effort to complete oral screening at least once or visit dental office for oral examination, preventive and prophylactic measures with oral hygiene instructions may be applied and ultimately require less treatment.

This is also related to your comment #2 regarding the longitudinal study. Regression model is meaningful and worthwhile to compare full-term and preterm infants in nationwide scale database, however, we did not find too many outcomes with significant difference from this study and thus decided regression model is not fit for this manuscript. If parents decide to leave survey questions blank or some items are left without reply, there exists missing values in the database. Database has multiple and various study participants and survey questions are simple and not specific enough to provide the exact present illness of patients and therefore, with lack of variables for comparison and without their significance, regression model is unfortunately trivial in this study. However, it will be very valuable in the future to set-up regression model using nationwide database cohort.

Revised text: This study found that the dietary habits and oral hygiene of preterm infants were predisposing factors for dental health problems in early childhood. However, controversially, rates of dental treatments such as behavior management, pulp treatment, or glass ionomer restoration were lower in preterm infants than in full-term infants. There were more preterm children who have never visited dental office. However, the average dental visit was similar between full-term and preterm children. For preterm born child who received at least one oral health screening service, the number of pulpectomy aiming to treat advanced dental caries that have invaded the dental pulp decreased. Therefore, this finding can be attributed to the fact that should preterm children make the effort to complete oral screening or visit dental office for oral examination, preventive and prophylactic measures with oral hygiene instructions may be applied and ultimately require less dental treatments in the future.

**Reply to reviewer #2**

**1. The authors concluded that preterm infants had poor oral health and dietary habits and fewer dental treatment experiences than full-term infants. There were many valuable variables collected in the nationwide cohort study. However, the authors merely presented the limited findings by a simple analytical fashion. In this retrospective study, readers may be interested in the differences in the potential factors for the poor oral health (as well as dietary habits or fewer dental treatment experiences) between the preterm birth and the full-term birth groups. The authors should perform the relevant regression analysis to explore them.**

Our response: Thank you for your valuable comment. I strongly agree with your opinion. We have not performed the regression analysis because Nationwide Infant Medical and Oral Screening Cohort Database(DB) includes limited variables related to oral health and substantial numbers of missing values as these data were not developed for research purposes but are conversion of medical and dental screening responses and claims. However, we believe that our results could be valuable for literature on oral health of children born preterm. Data extraction and minimal data set for this manuscript are explicitly recorded in reviewer 1 comment #1.

From this limited source, we have tried to pull out relevant variables and perform worthwhile analysis. We performed further analysis focusing on dentistry: we have looked at dental experience and treatment characteristics of preterm and full-term children and compared them per dental visit between two groups and also compared them based on completion of oral health screening. This is also related to reviewer 1 comment #8.

We modified Table 4 and added Table 5 and re-edited figure 2 to make this manuscript more meaningful. The following statements are take home messages from our further analysis:

1. There were more pre-term children who have never visited dental office.

2. However, the average number of dental visits were similar between full-term (5.94) and preterm (5.90) groups.

3. If a preterm-born child received at least one oral health screening service, the number of pulpectomies aiming to treat advanced dental caries decreased.

4. Therefore should pre-term children make the effort to complete oral screening at least once or visit dental office for oral examination, preventive and prophylactic measures with oral hygiene instructions may be applied and ultimately require less treatment.

Regression model is meaningful and worthwhile to compare full-term and preterm infants in nationwide scale database, however, we did not find too many outcomes with significant difference from this study and thus decided regression model is not fit for this manuscript. If parents decide to leave survey questions blank or some items are left without reply, there exists missing values in the database. Database has multiple and various study participants and survey questions are simple and not specific enough to provide the exact present illness of patients and therefore, with lack of variables for comparison and without their significance, regression model is unfortunately trivial in this study. However, it will be very valuable in the future to set-up regression model using nationwide database cohort.

Revised text: This study found that the dietary habits and oral hygiene of preterm infants were predisposing factors for dental health problems in early childhood. However, controversially, rates of dental treatments such as behavior management, pulp treatment, or glass ionomer restoration were lower in preterm infants than in full-term infants. There were more preterm children who have never visited dental office. However, the average dental visit was similar between full-term and preterm children. For preterm born child who received at least one oral health screening service, the number of pulpectomy aiming to treat advanced dental caries that have invaded the dental pulp decreased. Therefore, this finding can be attributed to the fact that should preterm children make the effort to complete oral screening or visit dental office for oral examination, preventive and prophylactic measures with oral hygiene instructions may be applied and ultimately require less dental treatments in the future.

**2. In this study, data on socio-economic variables, disability, and medical resource utilization status such as consultations and medical screening were collected; please show the information, which giving readers a clear feature regarding the study population.**

Our response: Thank you for your comment. We have revised table 2 which outlines baseline characteristics of the study population and added sex, household income and disability.

Revised text: The evaluation at 4-6 months when the first health screening was performed, the preterm group had a significantly lower household income of less than 30 percentiles (*p*=0.022). In addition, the proportion of children with disabilities in preterm birth was significantly higher (*p*=0.023), however, the overall number of children certified disability registrations was lower.

**3. Please place the numbers of the preterm birth and the full-term birth groups respectively in Tables 2, 3 and 4, which can make the relevant information clear and easy for readers to understand.**

Our response: Thank you for your comment. Unfortunately, Korean medical and oral health screening sessions do not match and therefore it is not easy to unite responses into undivided section. Table 2 and 3 have footnote indicating specific responses from oral health screening. Thus, it is not easy to separate columns by preterm and full-term birth groups and the table may become too complicated to comprehend. However, if it would still be more helpful to readers, please let us know and we would be happy to revise it. Table 4 is entirely based on oral health screening and thus, we have added the numbers of the preterm and full-term birth groups.

Revised text: We have added screen capture of part of Table 4 to show added numbers of preterm and full-term birth. (highlighted in yellow box)

**Reply to reviewer #3**

**1. The author's study shows that the oral hygiene-related characteristics of preterm baby are very bad and dangerous compared to full-term baby, and it would be better to show it in an arithmetically impactful schematic that is easier to understand.**

Our response: Thank you for your comment. Unfortunately, characteristics related to the oral hygiene are exclusively based on parents’ subjective opinion on their child’s oral hygiene and a simple yes or no question on detectability of visible dental plaque on teeth surface reported by dentists responsible for oral health screening. This is shown in Table 3. There are no objective indexes related to oral hygiene recorded in medical or oral health screenings.

**2. The current table and picture are not enough to show the results and conclusions. For example, the percentages listed in Figure 2 are intuitively difficult to understand.**

Our response: Thank you for your comment. We have revised table 2 which outlines baseline characteristics of the study population and added sex, household income and disability. We revised table 4 and added table 5 to improve comparing the dental experience of preterm birth and full-term birth. The average dental experience per 1 dental visit, the sum of each dental treatment per single dental visit, are added as the outcome variable. The dental experience and the average dental experience per 1 dental visit of those who had undergone an oral health screening at least once and those who had never had oral health screening are analyzed separately. We have also updated Figure 2 in hopes of showing better presentation of the summary of dietary habits, oral characteristics and dental treatments in preterm infants in NHSIC.

**3. There are some parts in the text that don't match the percentage. In a study using big data, accurate arithmetic expression without errors is essential to assert the characteristics of groups through representative values and present them as conclusions.**

Our response: Thank you for your critical comment. We found some mistakes in matching numbers and percentages. We went over the whole manuscript to make sure the manuscript is numerically unified.

**4. The comparison of oral characteristics of preterm baby and full term baby shown in Table 3 shows no significant difference in all cases except for '42-53 months of oral hygiene'. Considering the tone of the text that Preterm baby is not better, Table 3 seems to show the opposite results.**

Our response: Thank you for your thoughtful comment. There are two possible reasons for only 43-53 months of oral hygiene showing the significant difference in Table 3. First reason maybe because there are so little preterm born infants (*n* = 2374) compared to full-term born infants (*n* = 62525). In addition, tooth cavitation is usually the first visible sign for parents to recognize dental caries of their children. It takes months of time for a tooth to erupt through gingiva and for the crown to reach the functional occlusal surface. Thus visible caries can only be noticed by patients and/or parents only after some time after the beginning of tooth eruption. Primary teeth complete eruption in 24-30 months in average. The first oral health screening is done at 18-29 months and second at 42-53 months which is the most possible reason for preterm birth parents to report child’s poor oral hygiene at 42-53 months.

Revised text: It is difficult to observe caries at 18-29 months when primary molars start to erupt but have not fully erupted to the occlusal surface. Child’s oral hygiene becomes more noticeable to parents of preterm infants when tooth cavitation due to dental caries is recognized after the complete eruption of primary dentition. This is the underlying reason why preterm parents of 42-53 months significantly recognized the child’s oral hygiene to be poor. However, there was no significant difference between the two groups at the follow-up examination at 54-65 months.

**5. In addition, "food impaction", "poor oral hygiene", and "visible dental plaque" are all overlapping items, but they all marked differently and processed statistics separately, so please explain why they were classified separately.**

Our response: Thank you for your comment. “Food impaction”, “poor oral hygiene”, “visible dental plaque” are questionnaire items answered by the parents and included in the oral screening. They are conceptually overlapping however, they are separate items asked in each screening and this is why they were processed statistically separately. We have included explanations on how the item was extracted and elaborated on “poor oral hygiene” item as an example.

Oral health questionnaire answered by parents are not provided in English. We have selected parts of the questionnaire and included the original Korean version as well as the translated English (in blue font) for your better understanding.

Oral Health Screening Questionnaire

(This questionnaire is for 18-29 months)

Oral health screenings are available for 2Y(18 - 29M), 4Y(42 – 53M) and 5Y(54 – 65M). This survey is intended to help with dentist evaluation later. The data is later collected as personal ID and all personal information is classified.

Dental history and Parent’s report of child’s oral hygiene

1. Has your child visited a dental clinic for oral examination or dental treatment in a past year?

(1) yes (2) no

2. Has your child complained of dental/tooth pain in a past month?

(1) yes (2) no

3. Do you think your child currently has dental caries?

(1) yes (2) no (3) I don’t know

The same questions are asked in 2^nd^ (42–53 months) and 3^rd^ (54–65 months) sessions of oral health screening.

Like explained in the minimal data set section, data for this manuscript was collected from data provided by National Health Insurance Service(NHIS). NHIS has nationwide infant medical and oral screening cohort data base user manual but is not provided in English. We have also selected parts of the manual and included the original Korean version as well as the translated English (in blue font) for your better understanding. If you need further details, please refer to reviewer 1 comment #1 response.

| Number | Variable | Variable Code | Variable type | Note |
| --- | --- | --- | --- | --- |
| 32 | Rate your child’s oral hygiene | IO1Q_Q05_V0709 | NUM(8) | 1 Good  2 Moderate  3 Poor |
| 60 | Rate your child’s oral hygiene | IO2Q_Q05_V0709 | NUM(8) | 1 Good  2 Moderate  3 Poor |
| 93 | Rate your child’s oral hygiene | IO3Q_Q05_V0709 | NUM(8) | 1 Good  2 Moderate  3 Poor |

IO1Q mean 1st session, IO2Q 2nd, and I03Q 3rd session respectively; indicating same question is asked every session.

Like shown above, all items were answered by parents in the survey and provided to us in separate database code. This is why each overlapping items were processed differently and also processed statistically separately.

**6. "Parent-reported poor oral hygiene and food impaction between teeth (Table 3) at the age of 42–53 months was higher in the preterm group than in the full-term group (p < 0.05)."** => **In addition, according to Table 3 in this sentence, 'food impaction' does not show a significant difference, but it is stated that it shows a significant difference together.**

Our response: Thank you for your insightful comment. Because of your insightful comment, we realized this is a source of confusion as in original manuscript, both food impaction and poor oral hygiene seem to have significant difference. We have edited this statement for more clarification.

Revised text: Parents of preterm group reported higher food impaction between teeth and poor oral hygiene at the age of 42-53 months however, only the latter showed the significant difference (*p*=0.046).

**7. The results in Table 4 show that the full-term birth group as a whole received more dental treatment in most items than the preterm birth group. However, from these results, it may be interpreted that the full-term birth group is good at dental care, but it may be interpreted that the full-term birth group had more cavities and received more treatment**. **Therefore, it seems that the clinical data should be further supplemented for accurate analysis of Table 4.**

Our response: Thank you for thoughtful comment. Like you mentioned in your comment, if there are more treatments done in full-term group, this may come to a conclusion that full-term children’s oral is worse than preterm children. Thus, we have added analysis of number of dental treatments done per dental visit. There was a significant difference in number of dental visits between full-term and preterm children, however, number of dental treatments done per dental visit were not significantly different. This indicates that there is no significant difference in oral condition between two groups, especially if pre-term children made the effort to visit dental office for dental check-up and treatment. Table 4, 5, Figure 2 and the manuscript are edited accordingly.

We modified Table 4 and added Table 5 and re-edited figure 2 to make this manuscript more meaningful. The following statements are take home messages from our further analysis:

1. There were more pre-term children who have never visited dental office.

2. However, the average number of dental visits were similar between full-term (5.94) and preterm (5.90) groups.

3. If a preterm-born child received at least one oral health screening service, the number of pulpectomies aiming to treat advanced dental caries decreased.

4. Therefore should pre-term children make the effort to complete oral screening at least once or visit dental office for oral examination, preventive and prophylactic measures with oral hygiene instructions may be applied and ultimately require less treatment.

Revised text: The percentage of visits to the dentist at least once were significantly higher in full-term group (75.8%) than preterm group (74.0%) (*p*=0.036) (Table 4). However, the average number of dental visits for children were not significant between full-term (5.94±5.43) and preterm groups (5.90±5.31) (*p* = 0.783). Full-term children underwent 1 visit pulpectomy, pulpotomy and rubber dam application more often than the preterm children and required behavior management for less than 15 minutes. Full-term children had more glass ionomer restorations than preterm children (*p*=0.007) and preterm children had more sealants performed (*p*=0.034) when comparing dental treatment performed per dental visit. Table 5 lists comparison of number of dental treatments done according to oral health screening completion. There was no difference in dental treatments between full-term and preterm children if a child had never received oral health screening. For preterm children who have completed at least one oral health screening, 1-visit pulpectomy (*p* = 0.007) and 2-visit pulpectomy (*p* = 0.042) were performed less. Dietary habits and dental experiences of preterm children are summarized in Fig 2.

This study found that the dietary habits and oral hygiene of preterm infants were predisposing factors for dental health problems in early childhood. However, controversially, rates of dental treatments such as behavior management, pulp treatment, or glass ionomer restoration were lower in preterm infants than in full-term infants. There were more preterm children who have never visited dental office. However, the average dental visit was similar between full-term and preterm children. For preterm born child who received at least one oral health screening service, the number of pulpectomy aiming to treat advanced dental caries that have invaded the dental pulp decreased. Therefore, this finding can be attributed to the fact that should preterm children make the effort to complete oral screening or visit dental office for oral examination, preventive and prophylactic measures with oral hygiene instructions may be applied and ultimately require less dental treatments in the future.
