# Supplementary material for: Oral characteristics and dietary habits of preterm children: A retrospective study using National Health Screening Program for Infants and Children
Source: PLoS One. 2023 Mar 2;18(3):e0281896. doi: 10.1371/journal.pone.0281896 (PMC9980808; doi:10.1371/journal.pone.0281896)
Supplement: Attachment — Submitted filename: response to reviewer_minor revision_final230131.docx [file pone.0281896.s002.docx]

**A rebuttal letter to editor and reviewers**

Dear Editor and Reviewers,

We thank you for giving us the opportunity to make a minor revision. The authors tried to answer the reviewer's additional requests as sincerely as possible. I look forward to working with you and the reviewers to move this manuscript closer to publication in PLOS ONE. Thank you for your consideration. I look forward to hearing from you. Here is a point-by-point response to the reviewers’ comments and concerns. The reviewers’ comments are in bold. Our response follows and edited manuscript is highlighted in yellow.

**Editor Comments:**

**I think it would be better to present the statistics (results of regression analysis) and let the reader read and make their own judgment in respect of the study power of your work, instead of simply arguing that “we did not find too many outcomes with significant difference from this study and thus decided regression model is not fit for this manuscript.” In fact, it is kind of surprising to me with reference to the declared outcomes, considering it is a nationwide, population-based longitudinal study and the sample size is sufficiently large (N = 84,005).**

Our response: Thank you for your thoughtful comment. Like you and reviewer 2 suggested, we have included results of regression analysis in our revised manuscript and incorporated our thoughts and interpretation in the discussion as well. We fully respect the statistics criteria for publication at Plos ONE and we have put in effort to meet the standards. Once again, thank you for your advice and comments.

**Reply to reviewer #1**

**I am pleased to be in charge of the review of the revised paper. I have confirmed that very sincere responses and corrections have been made to the points pointed out by several reviewers. Also I confirmed that it was reflected in the revised manuscript. Despite the limitations of the study, this study contains meaningful data to publish and examines the relationship between low birth weight and oral health. Therefore, it is judged to be suitable for publication.**

Our response: Thanks for your response. All authors are delighted and honored that the revised manuscript can satisfy your request.

**Reply to reviewer #2**

**The authors still presented the limited findings by a simple analytical fashion in the revised manuscript. In this retrospective study, readers may be interested in the differences in the potential factors for the poor oral health (as well as dietary habits or fewer dental treatment experiences) between the preterm birth and the full-term birth groups. The authors can use the relevant regression analysis to explore them in the preterm birth and the full-term birth groups, respectively. Why did the authors NOT perform these regression analyses? Please explain it.**

Our response: Thank you for your valuable advice. We have constructed multivariable logistic regression on the association between preterm birth and receiving oral health screening. The model includes characteristics of study participants, medical history, and dietary habits as explanatory variables.

There is no absolute and objective factor like DMFT to represent oral health for comparison in the available study data. In our results, we have found that preterm born babies do not necessarily correlate to having more dental treatments, but actually, less or similar dental treatment experiences compared to full-term born groups and therefore it is difficult to conclude preterm birth is a predisposing factor for poor oral health.

In this study, the authors would like emphasize that preterm born children who have completed oral health screening have decreased dental treatment experiences, especially the need of pulpectomy which is done to treat severe dental caries or caries with lesion extended to periapical region. Therefore, the authors have included factors that could potentially affect oral health screening in the regression model.

The regression model depicted that the rate of completing oral health screening is higher in preterm born children than full-term born children which ultimately suggests that even though preterm born children have long bottle feeding period, low appetite and poor oral and dietary habits, those who put in the effort to complete oral health screening, can potentially attenuate the possibility of severe dental treatments through regular oral examination and oral health instructions.

Revised text:

**Study participants**

For dental treatment analysis, the remaining 66,373 (79%) infants were included. Among them, 27,200 infants who had not finished fifth infant health screening were excluded for multivariable logistic regression analysis on association of preterm birth and receiving an oral health screening. The regression model included 39,713 (47%) infants.

**Statistical analysis**

Multivariable logistical regression model and Wald test was used to evaluate an association between potential factors including preterm birth and completion of oral health screening. Birth history and demographic factors such as sex, disability and income were included to model and dietary habits were selected based on backward selection with at least marginal significance (p<0.2) on univariate analysis.

**Results**

**Preterm birth and oral health screening completion**

The multivariable logistic regression model included the potential variables shown in Table 6. The preterm birth group presented a significantly higher probability of oral health screening completion than the full-term birth group (aOR 1.141, 95% CI 1.015 to 1.283). Moreover, oral health screening completion was significantly higher in subjects who responded to breastfeeding at 4-6 months (aOR 1.094, 95% CI 1. 048 to 1.142) and vitamin supplements intake at 18-24 months (aOR 1.105, 95% CI 1.059 to 1.154). On the other hand, oral health screening completion was significantly lower in subjects who were male (aOR 0.956, 95% CI 0.917 to 0.997), had low household income (aOR 0.937, 95% CI 0.882 to 0.996), late start of weaning food in >6 months (aOR 0.901 95% CI 0.855 to 0.950), and breastfeeding at 18-24 months (aOR 0.846 95% CI 0.805 to 0.890).

**Table 6. Multivariable logistic regression analysis for oral health screening completion**

| Variable and classification | aOR | 95% CI of aOR | | *p*-value |
| --- | --- | --- | --- | --- |
|  |  | Lower | Upper |  |
| Birth history  Full-term birth (Ref.)  Preterm birth | 1.141 | 1.015 | 1.283 | 0.0269^*^ |
| Sex  Female (Ref.)  Male | 0.956 | 0.917 | 0.997 | 0.0366^*^ |
| Disabled  No (Ref.)  Yes | 0.754 | 0.125 | 4.541 | 0.7582 |
| Low household income (<30 percentile)  No (Ref.)  Yes | 0.937 | 0.882 | 0.996 | 0.0373^*^ |
| Breast feeding at 4-6 months  No (Ref.)  Yes | 1.094 | 1.048 | 1.142 | <0.001^*^ |
| Start of weaning food  ≤6 months (Ref.)  >6 months | 0.901 | 0.855 | 0.950 | 0.0001^*^ |
| sugar-added beverages at 18-24 months  < 200ml (Ref.)  ≥200ml | 0.954 | 0.880 | 1.035 | 0.2560 |
| Vitamin supplements intake at 18-24 months  No (Ref.)  Yes | 1.105 | 1.059 | 1.154 | <0.001^*^ |
| Breast feeding at 18-24 months  No (Ref.)  Yes | 0.846 | 0.805 | 0.890 | <0.001^*^ |

aOR, adjusted Odds Ratio

CI, Confidence Interval

Ref., Reference group

* Multivariable logistical regression model and Wald test, p<0.05.

**Discussion**

The available data from this study, unfortunately, could not provide index such as DMFT to accurately and objectively represent oral health. However, the regression model supports higher completion of oral health screening in preterm born group, more significantly higher if one was breastfeeding at 4-6 months and taking vitamin supplements at 18-24 months. On the contrary, preterm born children who started weaning food later than 6 months or were breastfeeding at 18-24 months had lower completion which can be attributed to the fact that, parents are less aware of the need of oral health screening because an obvious problem related to oral health is not yet visible.
